# Review of Welding Technologies for Electronic Precision Equipment: Status, Limitations, and Prospects

**DOI:** 10.3390/ma19183976

**Published:** 2026-09-19

**Authors:** Haoyu Cai, Renche Wang, Xingmei Feng, Huili Cao, Tao Wang, Huamin Yu, Molin Su

**Affiliations:** 1 Nanjing Research Institute of Electronics Technology, Nanjing 210039, China; 2 CNPC Research Institute of Safety & Environment Technology, Beijing 102206, China

**Keywords:** electronic precision equipment, welding, physical limitations, engineering breakthrough

## Abstract

This review examines the application of mainstream welding technology in electronic precision equipment, focusing on the matching relationship between the three dimensions of joint strength, spatial adaptability and welding deformation and the requirements of electronic precision equipment. The authors critically evaluated the application status and physical limitations of vacuum brazing, electron beam welding, friction stir welding and laser-arc hybrid welding in the field of electronic precision equipment. A criterion-based comparison table and a unified classification of the physical limitations are provided to support technology selection. On this basis, this paper further discusses the microstructure characteristics of each welding method and the regulation mechanism of key process parameters on joint performance. The review finally points out that the current engineering bottleneck is not the lack of connection ability, but the coupling constraints among thermodynamic constraints, material intrinsic physical properties constraints and spatial geometric contradictions, and proposes the expected direction of future engineering breakthroughs.

## 1. Introduction

In order to meet the operational requirements of high precision, high mobility and high reliability of electronic precision equipment in modern warfare, electronic equipment is further developed in the direction of high integration, lightweight and efficient heat dissipation. At the same time, with the development of various equipment for deep space, deep sea and ocean, electronic equipment is facing a more extreme working environment, such as high/low temperature, high humidity and high salt, complex electromagnetic environment, high impact and other extreme environments. On this basis, all kinds of new structures and complex precision structures appear in large numbers, which puts forward higher and higher requirements and challenges to advanced technology. At this time, a variety of advanced welding technologies have emerged, such as high-energy beam welding [1,2,3] (electron beam welding, laser welding), solid-phase welding [4,5,6] (friction stir welding), high-efficiency brazing [7,8,9] (vacuum brazing), multi-energy field composite welding [10,11,12] (laser-arc composite welding), etc., which can achieve high-reliability welding of complex structures and precision components, and become the key technical support for electronic equipment manufacturing.

In the field of electronic equipment, the application of advanced welding technology has promoted the improvement of equipment performance [13,14]. Taking phased array radars such as SPY-6 in active service of the US military as an example, the titanium alloy/aluminum alloy packaging structure of its T/R components is widely used in electron beam welding and vacuum brazing processes to achieve high weld strength and deformation. The technical requirements meet the stringent service requirements of shipboard/aviation equipment [15,16,17]. Laser welding and friction stir welding are widely used in the fuselage frame and wing structure of Boeing 787 aircraft, which take into account the technical characteristics of small deformation and high strength [18,19,20]. Narrow gap submerged arc welding and laser-arc hybrid welding are used to achieve efficient welding of 50 mm ultra-thick plates in large-scale structural skeletons and marine engineering equipment, ensuring structural flatness while taking into account structural integrity [21,22,23].

With the gradual deepening of the engineering application of phased array radar in land, sea, air and space platform weapons and equipment, and the continuous improvement of various technical indicators, the high integration of product structure leads to the increasingly tight welding space. The continuous improvement of equipment accuracy makes the deformation control requirements more stringent, and the lightweight trend makes the requirements of bearing components for mechanical properties continue to increase. For example, the flatness requirements of multi-layer cavity waveguides and micron-scale microchannel cooling components with wall thickness less than 0.8mm are extremely demanding, and precise connections need to be achieved by vacuum brazing and other technologies. After the weight reduction design, the high-load turntable needs to use high-strength and high-toughness materials to ensure the rigidity and strength. However, the characteristics of the material itself make it difficult for the traditional welding technology to meet the reliability requirements. It is necessary to meet the precision high-strength connection through laser-arc hybrid welding.

In this review, electronic precision equipment refers to complete electronic systems delivered as integrated platforms rather than to chip-scale devices alone. A vehicle-mounted phased-array radar, for instance, comprises two structural tiers: (i) the load-bearing skeleton (e.g., the deployable antenna frame), whose post-weld flatness must be maintained within 1 mm per 1 m to preserve the electronic signal accuracy of the array; and (ii) the precision functional devices (multi-layer cavity waveguides, sub-arrays, and antenna horns), which require sub-millimeter flatness and tight-tolerance joints. Because the dimensional stability of the skeleton governs the electronic signal accuracy of the array, the skeleton is itself a precision load-bearing structure, and the joining of both tiers falls within the scope of this review. The precision functional devices are predominantly 6xxx-series alloys (e.g., 6061/6063 for antenna horns and waveguides); the load-bearing skeleton is currently the 5xxx aluminum [24], with 7075 (7xxx-series) targeted for future lightweight designs; and lower-cost product variants adopt structural steels such as Q345 [25]/Q690. This selection is customer-driven: a lighter skeleton frees weight for additional precision devices and thus improves the electronic signal accuracy, so high-grade platforms adopt the lighter but costlier aluminum alloys, whereas lower-cost variants adopt steels.

The four technologies reviewed here—vacuum brazing, electron beam welding, friction stir welding, and laser-arc hybrid welding—are those currently deployed for joining the two structural tiers under the service conditions of electronic precision equipment, namely a service life of about twenty years, sustained elevated-temperature operation, and contact with corrosive cooling media. Under these conditions, diffusion bonding, resistance welding, ultrasonic welding, active brazing, and transient liquid phase bonding have not demonstrated the long-term reliability required of load-bearing and hermetic structural joints and are therefore not treated as representative technologies.

Although numerous studies have been reported, a systematic treatment of joining technologies for electronic precision equipment is still lacking. Existing reviews address individual processes (e.g., laser welding, friction stir welding, laser-arc hybrid welding) or dissimilar-material joining in general, but none has examined the representative technologies from the perspective of the physical constraints limiting each, nor established the correspondence between specific structure tiers and the appropriate technology. This review addresses this gap by (i) defining the two-tier structure of electronic precision equipment, (ii) analyzing four representative technologies under a unified three-category classification of physical limitations, and (iii) providing a criterion-based comparison table for technology selection.

## 2. Brazing

**Figure 1:**
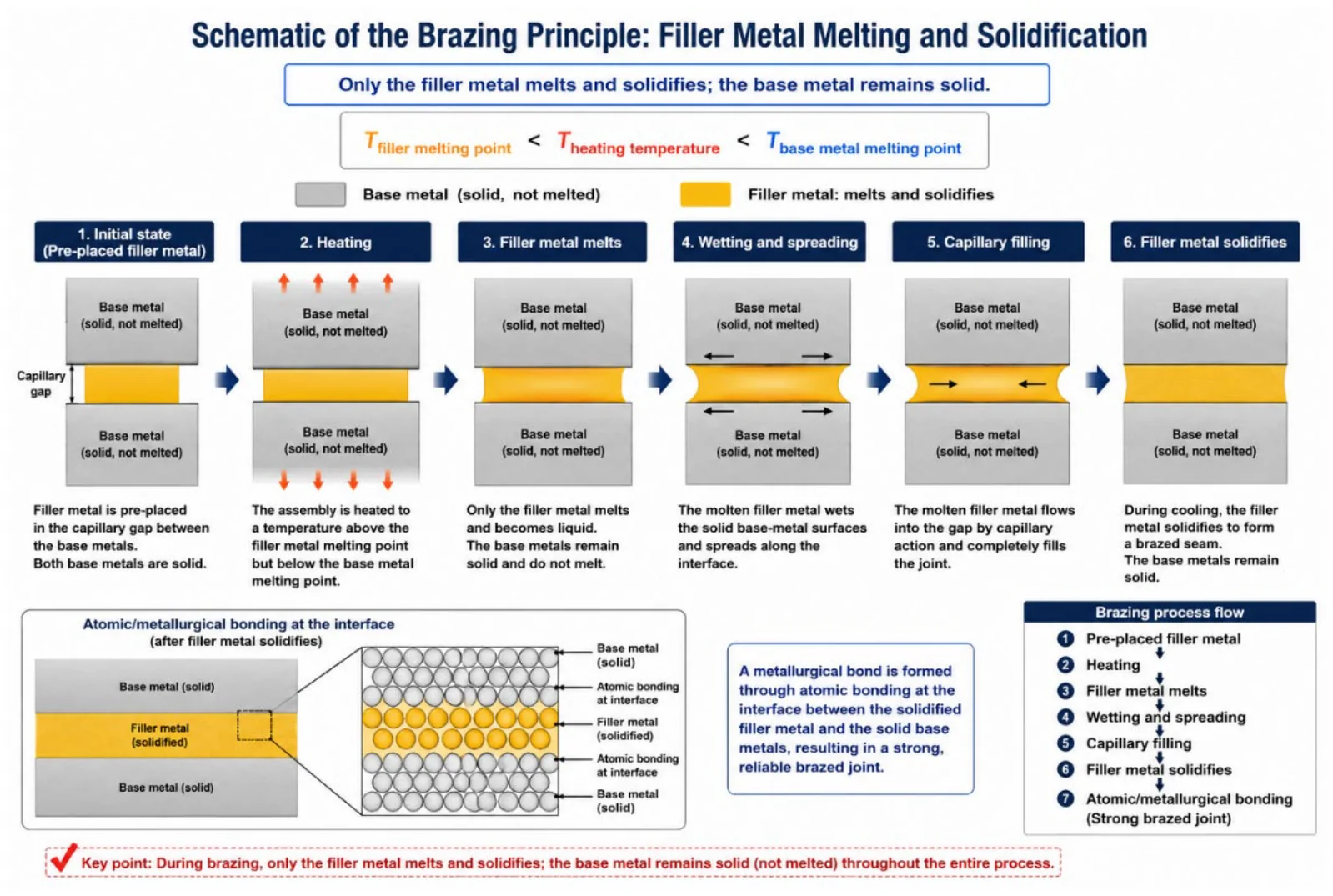
Schematic diagram of brazing [26].

The schematic diagram of the brazing is shown in Figure 1. The liquid solder is wetted, spreaded, capillary flowed and filled in the surface or gap of the base metal, and finally solidified and crystallized to achieve interatomic bonding.

### 2.1. Aluminum Alloy Brazing

Aluminum alloy electronic equipment has the following characteristics: (a) stringent deformation requirements, with the post-welding deformation controlled within 0.1 mm; (b) no gaps are allowed at the weld root, as root gaps will interfere with electrical signals; (c) limited operating space for welding. Given the above characteristics, aluminum alloys are not suitable for welding methods that induce large deformation, such as gas metal arc welding and friction stir welding. Although electron beam welding causes minor deformation, it entails relatively high costs and inevitably forms root gaps at the lap joints. In contrast, vacuum brazing can reduce costs by 75% and eliminate root gaps via rational lap joint design. Consequently, vacuum brazing is generally adopted for joining aluminum alloy components in electronic equipment.

**Figure 2:**
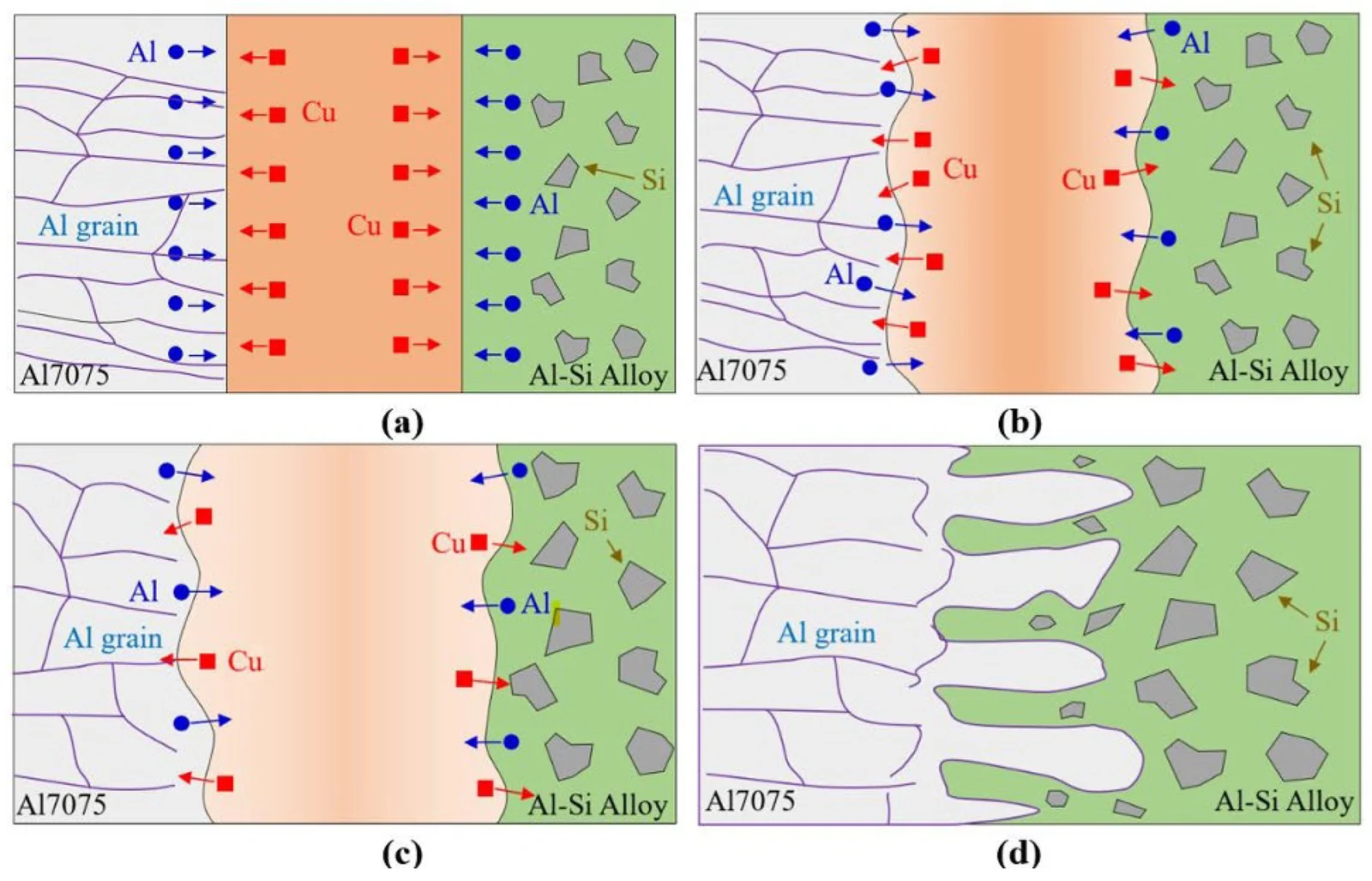
Schematic diagram showing formation mechanism of vacuum brazing joint between the dissimilar Al 7075 and Al-25 Si alloy: (**a**) solid-phase atomic diffusion reaction stage; (**b**) liquid phase generation and expansion stages; (**c**) liquid phase homogenization stage; (**d**) cooling stage [27].

Liu et al. [27] used 20 μm thick copper foil as the brazing filler metal to complete the vacuum dissimilar brazing of 7075 aluminum alloy and Al-25Si alloy at 560 °C for 10 min. The average shear strength of the brazed joint is 26.4 MPa. During heating below TE (548.2 °C), only mutual diffusion occurs between Al and Cu atoms. Since Cu atoms diffuse faster in Al matrix than Al atoms in Cu, massive Cu atoms preferentially migrate into the aluminum substrate and generate diffusion gradients, thereby forming various diffusion transition layers (Figure 2a). When the temperature exceeds TE, dissolved Cu reduces the melting point of α-Al by forming solid solutions. Correspondingly, eutectic liquid phase generates at the bonding interface. Continuous dissolution of Al and Cu gradually expands the liquid phase until the intermediate Cu interlayer is completely consumed (Figure 2b). As the temperature rises to TB (560 °C) and keeps for 10 min, Cu atoms diffuse toward both base metals and more Al elements dissolve into the liquid phase, further broadening the liquid zone. Meanwhile, atomic diffusion homogenizes liquid components and relieves internal concentration gradients (Figure 2c). Nevertheless, the adopted holding time is insufficient to realize full isothermal solidification. The residual eutectic liquid fails to complete isothermal solidification before cooling. Finally, the remaining liquid solidifies during cooling and generates AlCu intermetallic compounds (Figure 2d). The microstructure observation shows that the copper solder is completely dissolved, the joint interface is irregularly serrated, and good metallurgical bonding is formed between the base metal. Xia et al. [28] used Al-Si brazing filler metal to braze Cu/Al by vacuum brazing technology. The brazing seam with good performance was obtained at a brazing temperature of 590–610 °C, vacuum of 10–3 Pa and holding time of 5–10 min.

**Figure 3:**
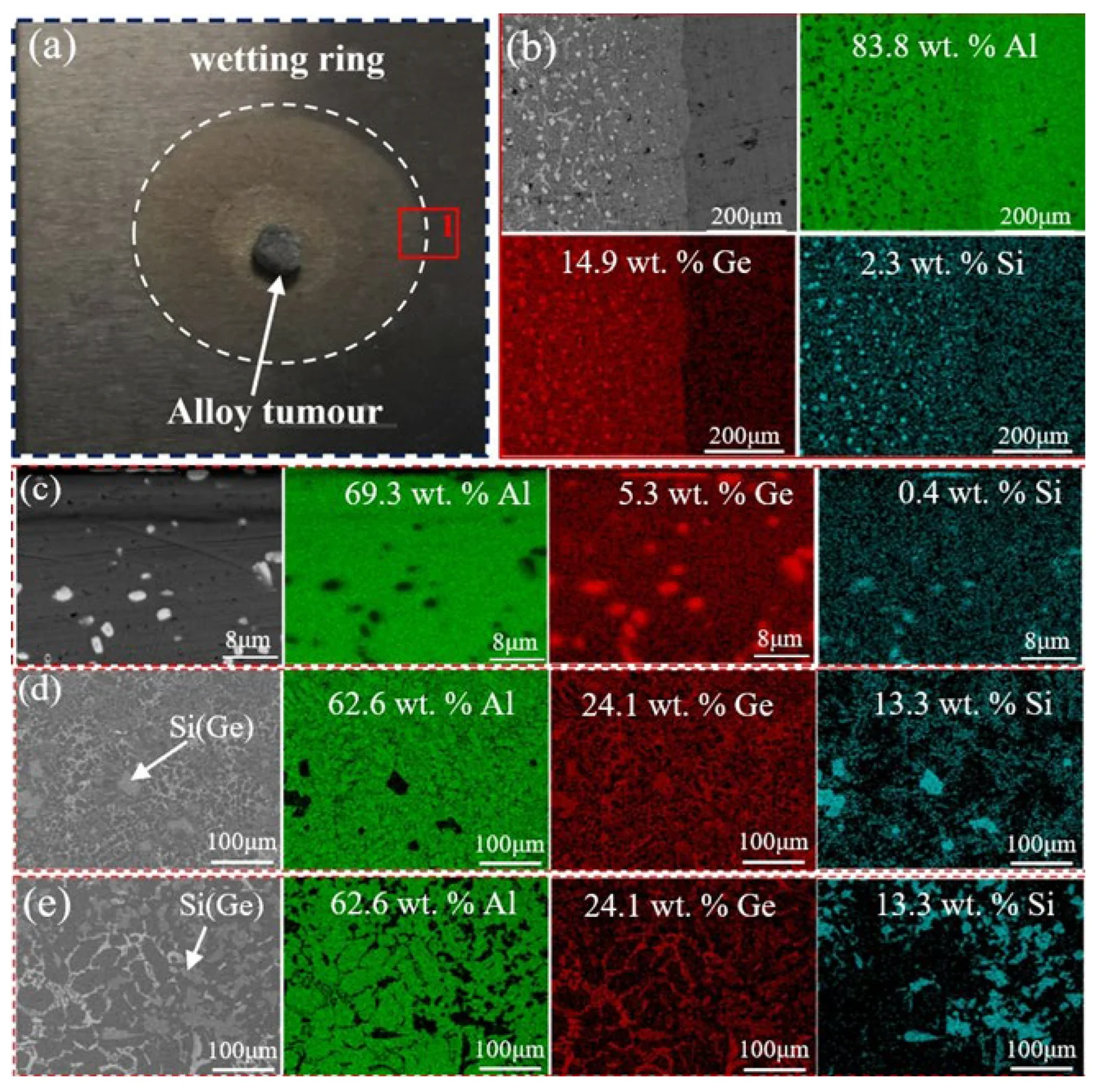
The wetting of Al-8Si-35Ge filler metal on the surface of the 6061 alloy: (**a**) wetting morphology; (**b**) the distribution of major elements of the wetting boundary marked with red line in the distribution of major elements of the cross-section of the wetting boundary; (**d**) original filler metal; and (**e**) residual filler metal [29].

Huang et al. [29] used Al-xSi-35Ge (x = 4, 6, 8, 10, 12, wt.%) solder to vacuum braze 6061 aluminum alloy and analyzed the mechanism and influence of Si in the microstructure of the brazed joint. The results show that the addition of Si has little effect on the melting point of Al-xSi-35Ge solder. Increasing the Si content in the solder can alleviate the liquefaction of the solder. This is because the chemical potential difference in Ge (μGe) in the solder and 6061 matrix decreases, which hinders the diffusion of Ge from the solder to the matrix, as shown in Figure 3. Du et al. [30] studied the effects of different solder thickness and different lap width on vacuum brazing of 6063 aluminum alloy antenna horn. It was found that the solder with more than 70% lap width can ensure that the brazing rate ≥ 80%, and the weld strength is about 75 MPa. The brazed joint strength is consistently 60–70 MPa (≈30% of the base metal) across the Al-Si, Al-Si-Ge, and 6063 antenna-horn studies, indicating that the brazed-joint strength is intrinsically limited by the filler metal.

The above findings demonstrate that the wettability of filler metal on the base material and the resulting metallurgical bonding quality can be effectively improved by tailoring the brazing process parameters and filler metal composition. Nevertheless, substantial gaps remain in the engineering application of two specific brazing routes.

The first concerns the brazing of die-cast aluminum alloys. The solidus temperatures of conventional die-cast aluminum alloys such as ADC12 and A380 fall within the range of approximately 515–545 °C. In contrast, the Al-Si eutectic filler metals commonly employed for aluminum brazing melt at around 577 °C, with the actual brazing process typically conducted at 590–610 °C. Consequently, the die-cast substrate enters a state of incipient melting before the filler metal can melt and wet the surface, rendering the joint incapable of meeting the fundamental requirement of substrate integrity during brazing.

The second involves the brazing of aluminum alloys to high-thermal-conductivity non-metallic materials. As electronic equipment continues toward higher levels of integration, the demand for heat dissipation has become increasingly stringent. Conventional metallic heat-dissipation materials, such as 6063 aluminum alloy, are approaching their intrinsic thermal conductivity limits. There is therefore an urgent need to introduce high-thermal-conductivity non-metallic materials—for example, AlN, SiC, and diamond/aluminum composites—and to join them to aluminum alloys through brazing, thereby achieving a step-change improvement in heat dissipation capability. However, critical issues in this direction, including interfacial wetting and thermal expansion mismatch, remain unresolved, and relevant studies have been scarcely reported.

### 2.2. Copper Alloy Brazing

Although aluminum instead of copper has become a trend in the electronic equipment industry, copper is still an excellent conductor, which can minimize the current loss of electromagnetic waves on the tube wall, thereby reducing signal attenuation. Copper waveguide is still a key component of electronic equipment. Because copper waveguide and aluminum waveguide have strict requirements on deformation, copper waveguide is also not suitable for large deformation welding methods (such as gas shielded welding, friction stir welding, etc.). For the electron beam welding with less deformation, the zinc element with lower melting point in the copper alloy will evaporate to form steam, which will pollute the cleanliness of the electron beam vacuum chamber. Therefore, copper alloys in the field of electronic equipment are usually welded by brazing.

**Figure 4:**
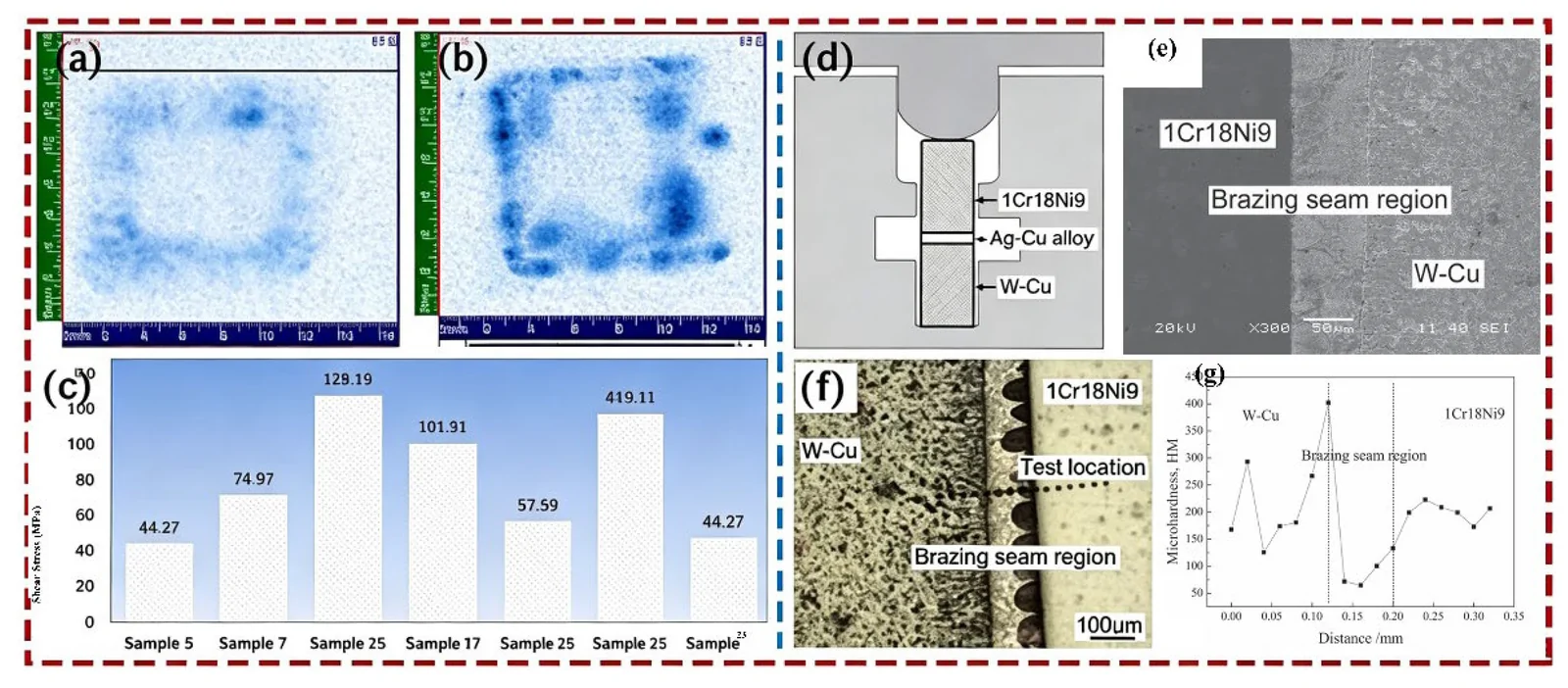
Heterogeneous connection of WCu/CuCrZr and WCu/stainless steel [31].

Owing to the superior properties of copper, its heterojunctions with dissimilar materials enable a broad range of applications in electronic equipment, principally thermal management and hermetic packaging-joining high-thermal-conductivity materials (W-Cu composites, graphite) or ceramic packages to metallic carriers. Xia et al. [31] performed vacuum brazing of WCu alloy and 1Cr18Ni9 stainless steel using Ag-28Cu filler metal, with a brazing temperature of 855–865 °C and a holding time of 30 min. An Ag-Cu eutectic phase was formed on the WCu composite side, which exhibited lower microhardness compared with the steel-rich phase on the 1Cr18Ni9 side. Cu and a small amount of W diffused from the WCu composite into the brazing seam, while Ag from the filler metal also diffused into the WCu alloy matrix, as shown in Figure 4. Four-point bending tests showed that the average bending strength of the joints reached 576 MPa, and fracture occurred on the WCu alloy side, indicating reliable bonding quality. Wang et al. [32] found that adding an appropriate amount of SiC particles into AgCuTi filler metal can further optimize the microstructure and mechanical properties of W/AgCuTi/Cu brazed joints. The joint strength reaches 119 MPa when the SiC content is 10 wt.%.

**Figure 5:**
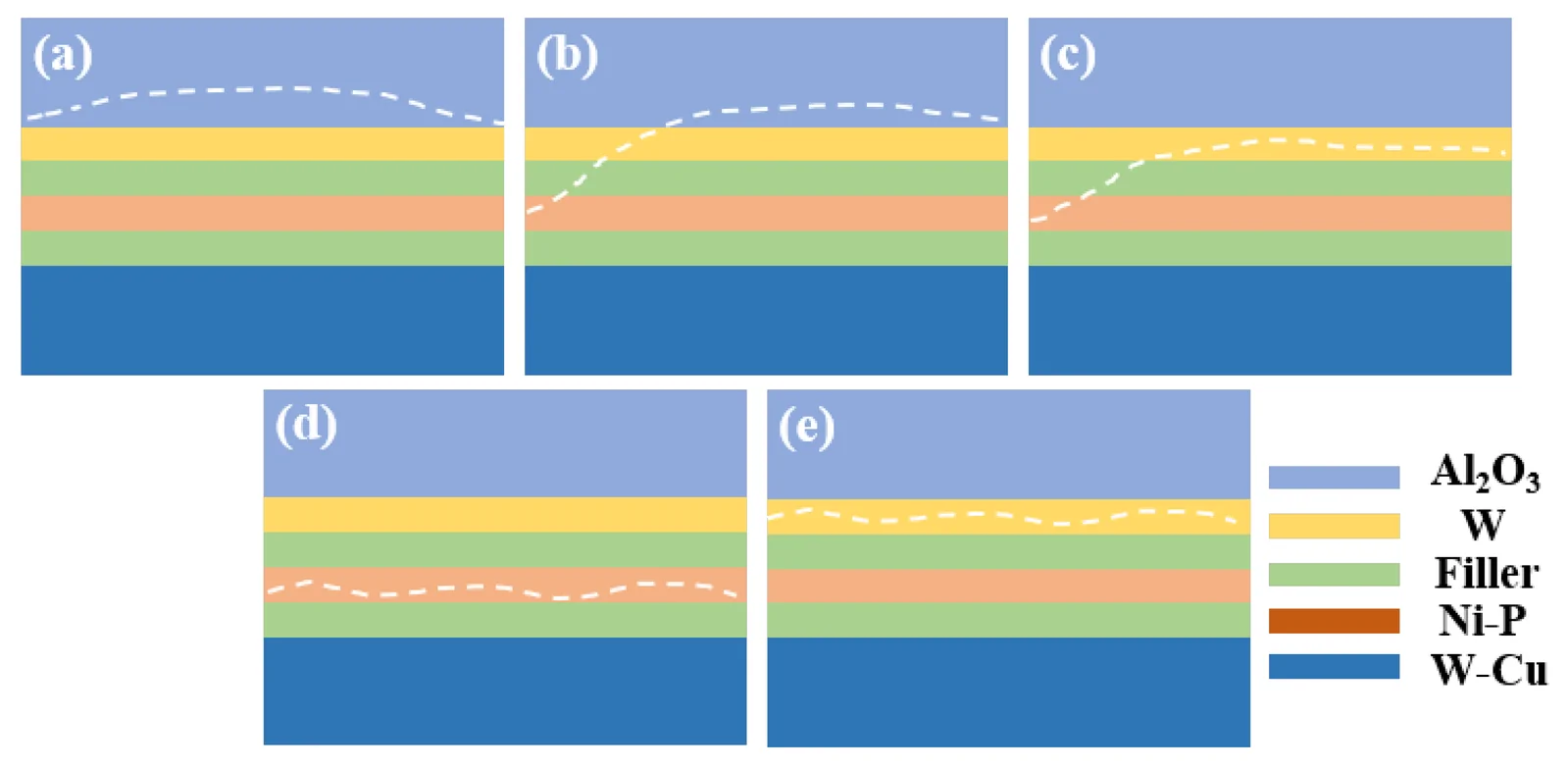
The schematic illustrations of fracture paths of W-Cu/HTCC joints.

Due to the substantial mismatch in both the coefficient of thermal expansion and the elastic modulus between ceramics and W-Cu alloys, achieving a reliable bond between the metallic heat-dissipation material and the ceramic substrate during the packaging process has become one of the critical challenges that must be overcome. Liu et al. [33] deposited Ni coatings on the surfaces of WCu alloy and high-temperature co-fired ceramic (HTCC) and adopted AgCu alloy as the filler metal to successfully fabricate WCu/Ni/AgCu/Ni/HTCC composite joints. The interfacial microstructure was composed of multiple phases, including Ni(Cu)-W, Ni_3_P, Cu-Ni, Cu solid solution, and Ag solid solution. The joint achieved a maximum shear strength of 74 MPa at a brazing temperature of 840 °C, whereas the minimum shear strength of only 30 MPa was obtained when the holding time was extended to 30 min. The thermal expansion coefficient of Al_2_O_3_ (6.8 × 10^−6^/K) is approximately 1.5 times that of tungsten (4.6 × 10^−6^/K), resulting in a smaller shrinkage on the W side than on the Al_2_O_3_ side. Accordingly, prolonged thermal holding at elevated temperatures induces the expansion of the HTCC substrate, generating compressive stress on the W side and tensile stress on the Al_2_O_3_ side. Numerous inherent pores inside the W layer act as crack initiation sources under stress loading, eventually leading to fracture failure within the W layer of the joints, as shown in Figure 5.

Among Ag-based filler metals, Zhang et al. [34] used Ni-Cr-P-Cu as the brazing filler metal to realize the brazing of graphite and Cu, and the obtained joint structure was graphite/Cr7C3 + Cr3C2 layer/Ni3P + Cr-C compound + Cu(s, s)/Cu. As the brazing temperature increased, the overall thickness of the brazing seam decreased, whereas the thickness of the reaction layer at the graphite--filler metal interface rose continuously. Mao et al. [35] realized the brazing of graphite and copper by using Cu50TiH2 brazing filler metal. The joint has no obvious metallurgical defects and is mainly composed of Cu (s, s) and Ti-Cu compounds. Appendino et al. [36] developed an indirect brazing route for joining graphite and copper alloys. Transition metals including Mo, W and Cr were first reacted with graphite to form metallic carbide coatings on graphite surfaces, followed by brazing copper alloys onto the pre-metallized graphite. The maximum shear strength of joints fabricated by this method reached 30 MPa. 

**Figure 6:**
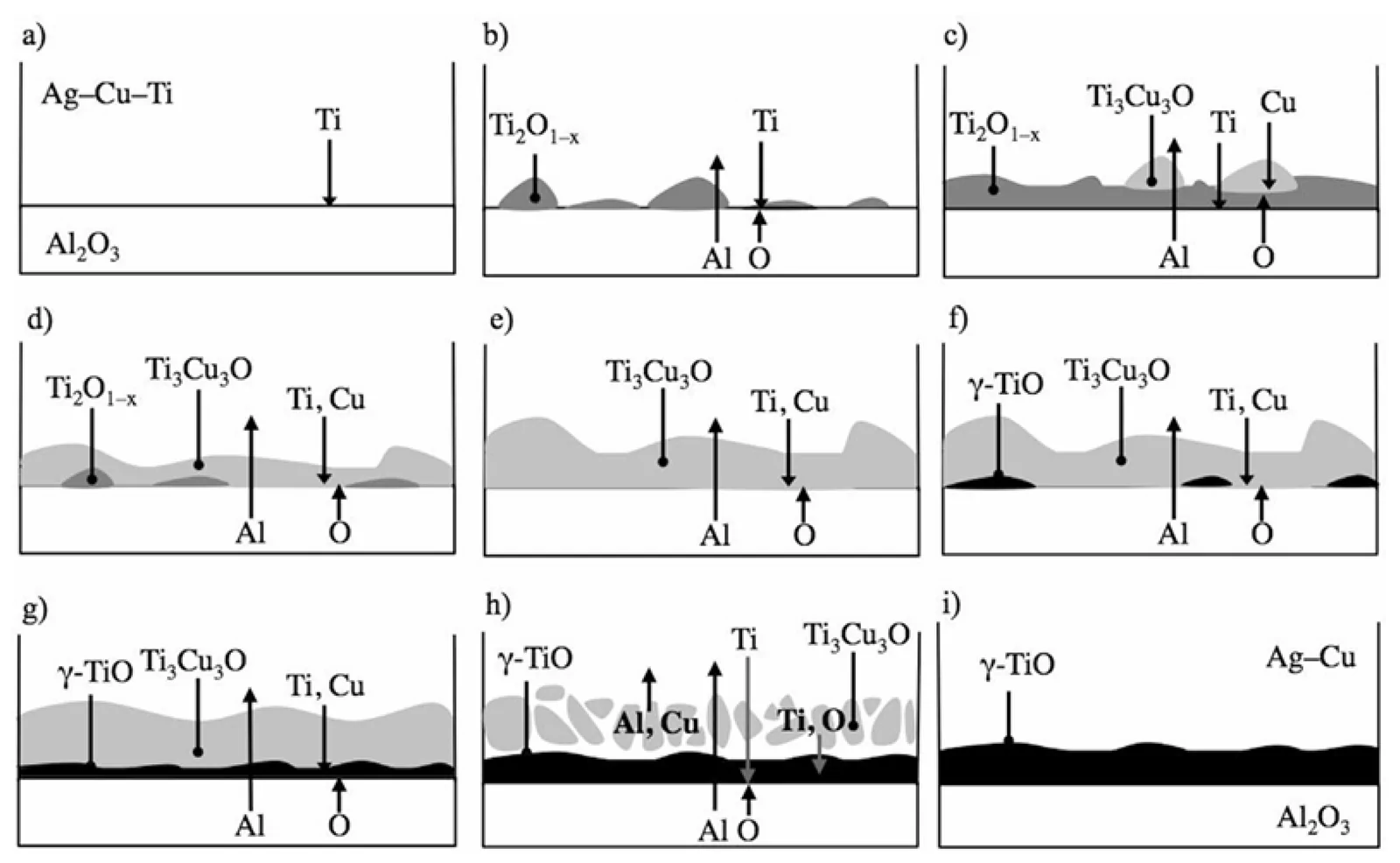
(**a**–**f**) Schematic mechanism for the evolution of interfacial phases at a Ag-Cu-Ti/sapphire interface during brazing, (**g**,**h**) along with the effect on the interfacial microstructure of holding at the peak temperature for extended periods of time [37].

Ali et al. [37] investigated the interfacial evolution mechanism during the brazing of sapphire with Ag-Cu-Ti active filler alloys. Active Ti elements in the liquid filler initially underwent metallurgical reactions with the sapphire surface to form a nanoscale polycrystalline layer. Afterwards, reaction particles nucleated and grew on the polycrystalline layer to form a continuous micron-thick layer, which further reacted with Ti and Cu atoms in the liquid filler in a secondary interfacial reaction, ultimately generating the ternary intermetallic compound Ti_3_Cu_3_O, as shown in Figure 6. Shen et al. [38] realized induction brazing of dissimilar joints between WCu alloy and beryllium bronze (QBe) using BAg56CuZnSn, BAg50ZnCdCuNi and BAg49ZnCuMnNi silver-based filler metals. Solid solutions such as Cu(Ag, Zn) and Ag(Cu, Zn) form within the brazed seam. Doping Mn into the filler metal homogenizes elemental distribution and significantly enhances grain refinement strengthening in the weld zone. Co-doping with Ni and Mn elements enables the joint strength to reach 250 MPa.

The above studies indicate that high-quality brazed joints between Cu alloys and various dissimilar materials including stainless steel, graphite and ceramics have been experimentally verified by regulating filler metal compositions, introducing active elements and conducting interfacial metallization pretreatment. The corresponding joint strengths can reach hundreds of megapascals, accompanied by excellent thermal cycle stability. Nevertheless, two critical engineering bottlenecks remain to be urgently addressed in this research field.

The first is the inherent tension between high joint performance and low material cost. The brazing systems that currently enable reliable copper-based joints rely heavily on Ag-based filler metals (e.g., Ag-Cu-Ti and BAg-series alloys), whose high silver content imposes a prohibitive material cost. Reducing the Ag fraction typically comes at the expense of wettability and joint strength. While strategies such as nanoparticle incorporation (e.g., SiC) and alloying element additions (e.g., Mn, Ni) have been shown to enhance the mechanical properties of joints at a given Ag content, they do not address the fundamental issue of precious-metal dependency. Developing novel filler metal systems that simultaneously achieve low Ag content and reliable metallurgical bonding represents the critical pathway toward cost-viable copper brazing at an engineering scale.

The second is the control of residual stresses in ceramic-metal heterojunctions during brazing. Taking the W-Cu/HTCC package as an example, the coefficient of thermal expansion of Al_2_O_3_ (6.8 × 10^−6^/K) is approximately 1.5 times that of tungsten (4.6 × 10^−6^/K), and the two materials differ substantially in elastic modulus. During post-brazing cooling, the thermal expansion mismatch generates tensile stresses on the ceramic side and compressive stresses on the metal side. Inherent pores within the tungsten layer act as crack initiation sites under these stresses, ultimately causing brittle fracture within the W layer at shear strengths of merely 30–74 MPa. The design of interfacial stress-buffer layers and graded thermal-expansion transition structures represents a key pathway to overcoming this barrier, yet research in this direction remains insufficiently developed.

## 3. Electron Beam Welding

**Figure 7:**
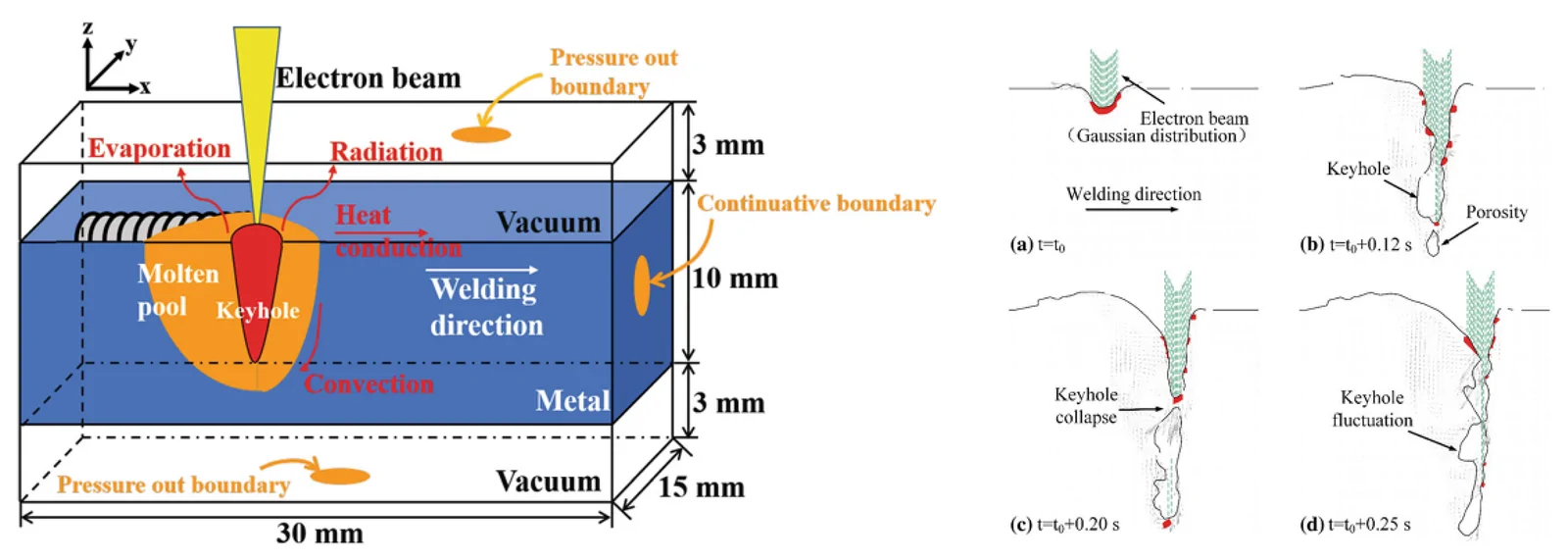
Energy conversion and deep penetration behavior of molten pool after electron beam bombardment on workpiece [39].

Although most of the aluminum alloy welding in the field of electronic equipment is mainly vacuum brazing, the tensile strength of the brazed joint is only about 60–70 MPa, which does not meet the needs of some aluminum alloy electronic equipment. For certain precision electronic equipment, the strength of the welds is required to exceed 80% of that of the base metal, accompanied by extremely narrow welding space and ultra-strict deformation tolerance, which necessitates welds with a large depth-to-width ratio. Restricted by the compact welding space, friction stir welding is unavailable for such structures, and EBW serves as a feasible alternative. During the EBW process, continuous interruption and reconstruction of the electron beam channel maintain a stable deep-penetration effect, which terminates once the welding system reaches dynamic equilibrium. The energy conversion mechanism and sustained deep-penetration behavior of the electron beam are displayed in Figure 7. With the relative movement between the electron beam heat source and the workpiece, the liquid molten metal flows rearward along the keyhole periphery and finally solidifies, forming welds with an ultra-high depth-to-width ratio. Electron beam welding possesses prominent advantages of strong penetration capability (weld depth ≥ 20 mm, depth-to-width ratio ≥ 8:1) and a narrow heat-affected zone. It effectively limits the welding deformation to 0.05 mm per 100 mm weld length, satisfying the rigorous precision demands of aluminum alloy electronic devices.

**Figure 8:**
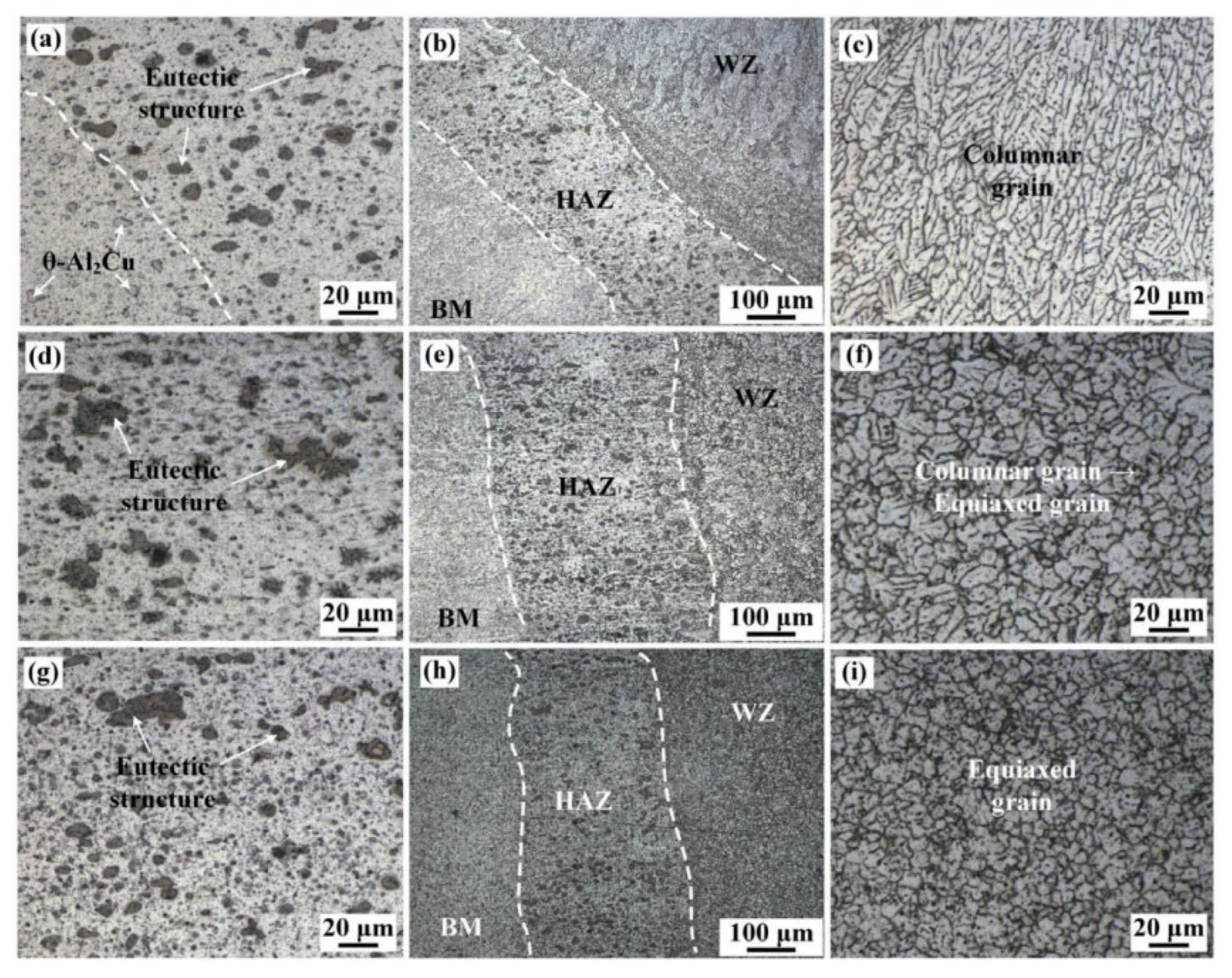
Metallographic microstructure of specimen #4: (**a**–**c**) upper, (**d**–**f**) middle, (**g**–**i**) bottom regions of 2219 joint, including the WZ (**c**,**f**,**i**), HAZ (**b**,**e**,**h**), and magnified HAZ (**a**,**b**,**g**) [43].

Cam et al. [40] conducted electron beam welding on 2024, 5005 and 6061 aluminum alloys, respectively, and explored the correlation between microstructure and mechanical properties of the welded joints. The experimental results demonstrate that 6061 aluminum alloy joints exhibit an obvious heat-affected zone (HAZ). Benefiting from the highly concentrated heat input of electron beam welding, strengthening precipitates in the weld metal are fully dissolved; hence the weld zone presents the minimum hardness across the joint. In addition, both 2024 and 6061 aluminum alloy welded joints show inferior tensile strength and ductility compared with the base metals, while 5005 aluminum alloy joints possess relatively better ductility. Qiao et al. [41] performed vacuum electron beam welding on 10 mm—thick 6061 aluminum alloy and characterized the mechanical properties of the welded joints. By optimizing key process parameters including electron beam current, focusing current and welding speed, joints with sound weld formation and superior comprehensive mechanical properties were obtained. Chang et al. [42] investigated the microstructure and mechanical properties of electron beam welded joints of 6061-T6 aluminum alloy. The test results indicate that the tensile strength of welded joints is lower than that of the base metal, and tensile fracture always occurs at the weld zone, which proves that the weld metal acts as the weak region of the whole joint. In addition, the joint possesses a narrow HAZ. Compared with the weld zone, the heat-affected zone contains more strengthening precipitates, thus presenting a slighter softening degree. Either pre-weld preheating or post-weld remelting treatment will induce obvious grain coarsening in the weld metal, which further reduces the tensile strength, microhardness and ductility of welded joints. Among all mechanical indicators, the ductility of joints is most significantly deteriorated. Cam et al. and Chang et al. consistently observed that 6061 EBW joints are weaker than the base metal, with fracture in the weld zone, whereas Qiao et al. showed that optimizing the beam current, focusing current, and welding speed yields sound joints with good comprehensive properties; the degree of joint softening is thus strongly process-dependent. Chang et al. [43] fabricated aluminum alloy joints by EBW. As the distance from the upper surface increased, the WZ microstructure near the fusion line transitioned from columnar grains to equiaxed grains, as shown in Figure 8c,f,i. The microstructure of the HAZ is presented in Figure 8b,e,h, in which a small number of black precipitates were formed, with a coarser size than that in the BM. This observation accounts for the higher strength of the bottom weld joint than that of the top joint.

Yang et al. [39] investigated the molten pool flow characteristics of electron beam welds. In the EBW of aluminum alloys, due to the molten pool behavior and high solidification rate, spiking defects tend to be generated at the weld seam bottom. With the oscillation of the molten pool, the formation of spiking defects also has a clearly periodic nature. The spiking defects and penetration depth fluctuations are inseparable and nearly simultaneous. In addition, Yang et al. [44] also studied the flow characteristics of the molten pool in electron beam circular welding. The research results show that limited by the supporting effect of base metal, the keyhole evolution presents a slight difference between flat welding and annular welding. After the welding process enters the full penetration stage, a tail-like structure is formed on the surface of the annular weld molten pool under the combined action of the Marangoni effect and recoil pressure. 

The studies reviewed above demonstrate that the weld pool morphology can be effectively regulated and the penetration depth increased by optimizing the matching between the electron-beam heat source characteristics and the welding parameters. The modulated energy input of the electron beam exerts a stirring effect on the molten pool, promoting the transition from columnar to equiaxed grains in the vicinity of the fusion line and thereby enhancing the joint quality. However, electron beam welding is by no means free of limitations. Constrained by the size of the vacuum chamber, stringent requirements on joint fit-up, and the cracking susceptibility induced by high cooling rates in certain alloy systems, its application to 7xxx-series aluminum alloys remains challenging. The wide solidification range of these alloys, combined with the pronounced evaporation of Zn and Mg under high vacuum, leads to compositional shifts in the weld metal, an increased tendency for hot cracking, and joint softening. These issues have yet to be systematically addressed, which currently restricts the engineering deployment of electron beam welding in this alloy class. Although electron beam welding is technically applicable to 6xxx-series aluminum alloys via filler-wire EBW, its high equipment and operating cost mean that, in engineering practice, structural steels are preferentially joined by laser-arc hybrid welding (Section 5) and 6xxx-series aluminum alloys by vacuum brazing (Section 2).

## 4. Friction Stir Welding

Friction stir welding (FSW) is suitable for aluminum alloy electronic equipment that requires high weld strength and offers relatively ample assembly space. FSW not only has wide material adaptability and can weld all types of aluminum alloys, but also has high-quality welded joints. Solid-phase welding avoids the melting process and eliminates defects such as pores and cracks. The tensile strength of the joint reaches 80% of the strength of the base material, which meets the requirements of GJB 294 A II weld. Most importantly, the cost of friction stir welding is extremely low, with only 8–12% of electron beam welding on a per-meter processing basis for comparable joint lengths (excluding workpiece-specific tooling amortization). 

In the process of FSW, the evolution of microstructure has an important influence on the mechanical properties of weld. Understanding the microstructure evolution mechanism of the material during FSW can help optimize the welding process and improve the weld quality. In recent years, the research on the microstructure evolution of FSW joints has received extensive attention at home and abroad, especially in the application of lightweight materials such as aluminum alloys. 

**Figure 9:**
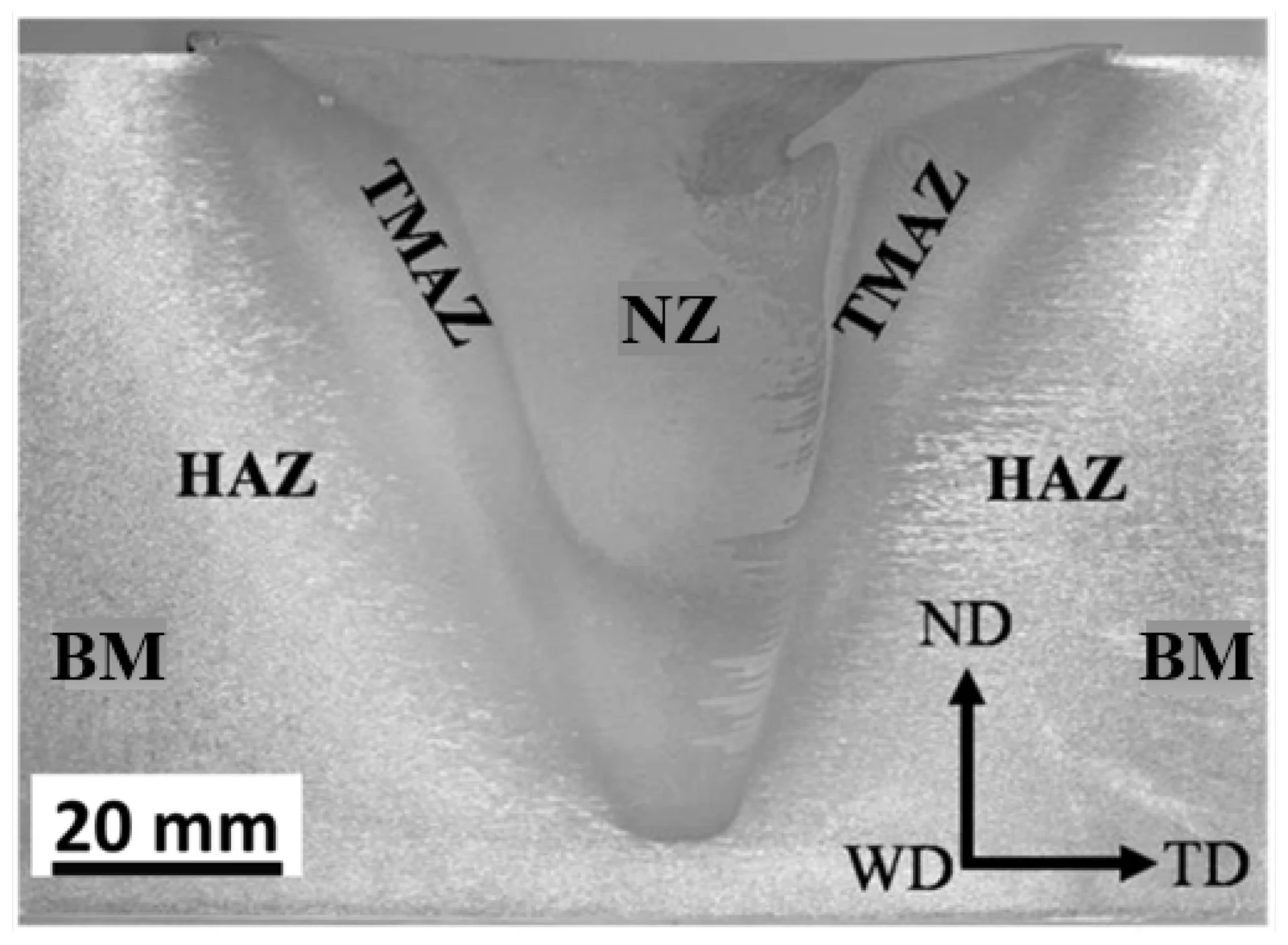
Division of FSW welded joint [20].

Due to the strong plastic effect of the stirring head and the high-temperature heat input, different microstructure characteristics are formed in the weld and its vicinity. The microstructure of the FSW joint is divided into four different regions from the center outward, namely the nugget zone (NZ), the thermo-mechanically affected zone (TMAZ), the heat-affected zone (HAZ) and the base material (BM), as shown in Figure 9 [20]. The solid-state properties and the gradient of strain, strain rate and temperature during FSW lead to highly characteristic microstructures.

Jacquin et al. [45] pointed out that the microstructure evolution during FSW, including grain size and grain boundary migration and orientation changes, directly affects the hardness and tensile strength of the joint. Grain refinement helps to improve the yield strength and toughness of the joint. During the grain boundary migration process, when the grain boundary orientation reaches a critical angle (about 15°), the small-angle grain boundary will be transformed into a large-angle grain boundary, thereby improving the mechanical properties of the material. Saluja et al. [46] used the Cellular Automaton (CA) method to simulate the effect of grain orientation distribution on the mechanical properties of FSW weld zone. Studies have shown that the formation of fine, randomly oriented grains in the weld zone helps to increase the yield strength and tensile strength. At the same time, the grain boundary with a large orientation difference (high-angle grain boundary) is particularly significant for the improvement of tensile strength. Abolusoro et al. [47] studied the relationship between process parameters, temperature, mechanical properties and microstructure during FSW of dissimilar aluminum alloys. It was found that an increase in temperature led to a change in microstructure in the welding area, and the precipitation of the second-phase particles led to a decrease in grain bonding force, which reduced the tensile strength of the weld. Zhu et al. [48] explored the temperature distribution, microstructure and mechanical properties of 6082 aluminum alloy during FSW. It was found that the grain size of different regions of the joint was different due to the influence of different temperatures, and the grain size of the heat-affected zone and the thermo-mechanically affected zone was coarsened, resulting in a decrease in hardness.

**Figure 10:**
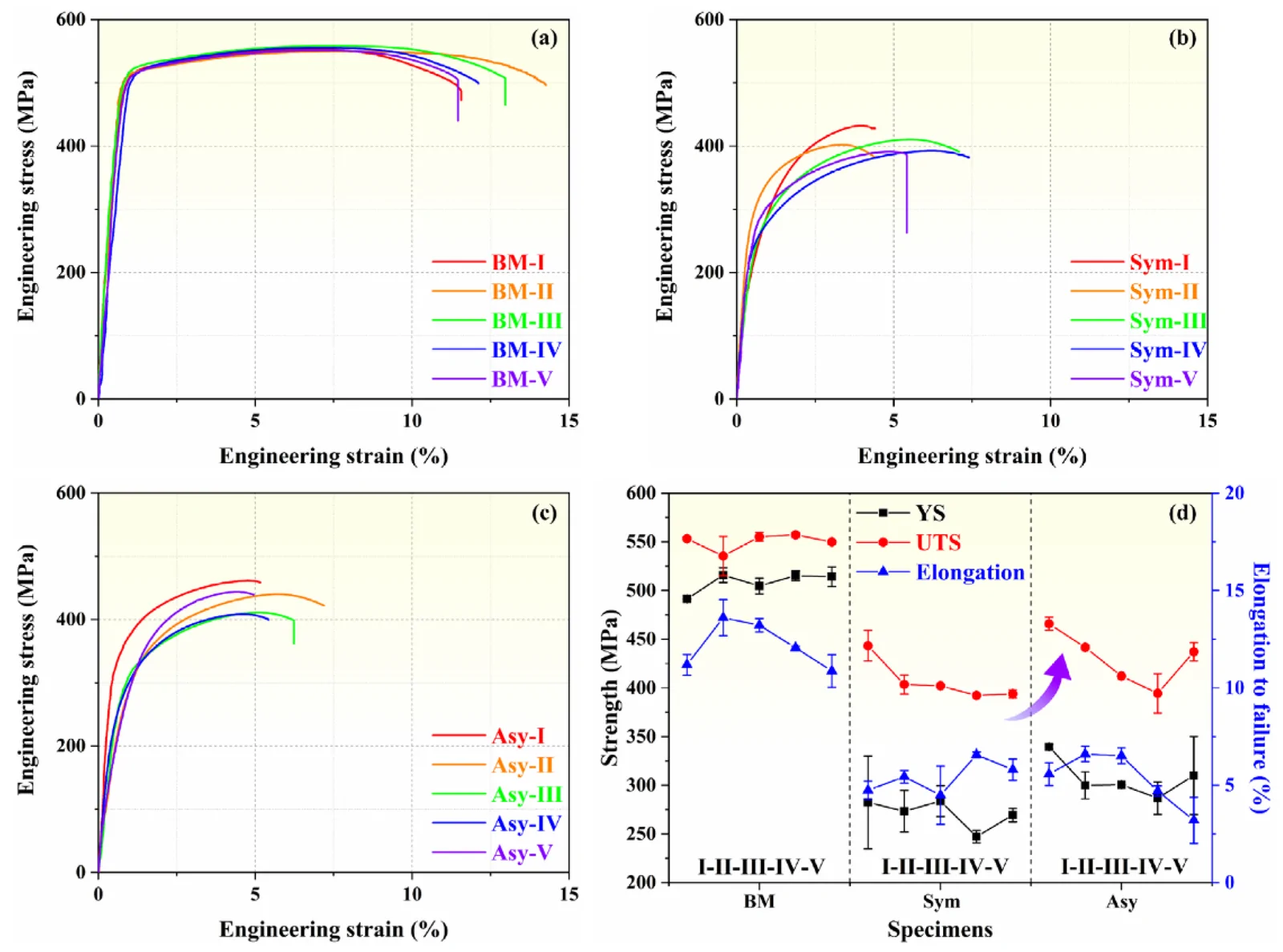
Tensile properties of BM and joints engineering stress-engineering strain curves of (**a**) BM, (**b**) Sym joints, and (**c**) Asy joints, (**d**) the comparison of the tensile properties [51].

Li et al. [49] carried out shoulder friction stir welding (S-FSW) experiments on 5 mm-thick AA6061-T6 aluminum alloy plates to investigate the effects of welding parameters on the microstructure evolution and mechanical properties of welded joints. The results showed that the joint achieved the maximum tensile strength of 256 MPa at a rotational speed of 1500 r/min and a welding speed of 300 mm/min. Chen et al. [50] comparatively studied conventional friction stir welding (C-FSW) and S-FSW for butt joints of 7075 aluminum alloy. Compared with C-FSW joints, S-FSW joints exhibited superior tensile strength. All friction stir welded joints failed in the nugget zone due to the existence of hook defects. The sharp-cornered hook defects further deteriorated the ductility of C-FSW joints, whose ductility was only 70% that of S-FSW joints. Across the alloys studied, the FSW joint strength exceeds 80% of the base metal (e.g., 256 MPa for AA6061-T6), and stationary-shoulder FSW outperforms conventional FSW for 7075 owing to less severe hook defects, highlighting the role of tool design in the achievable joint efficiency. Chen et al. [51] proposed an asymmetric double-sided friction stir welding (Asy-DS-FSW) and fabricated aluminum alloy FSW welded joints. The ultimate tensile strength of the slices is enhanced by 0.5–11% (Figure 10), and the eased strain localization can be achieved by the Asy-DS-FSW, compared with the conventional symmetric DS-FSW (Sym-DS-FSW).

**Figure 11:**
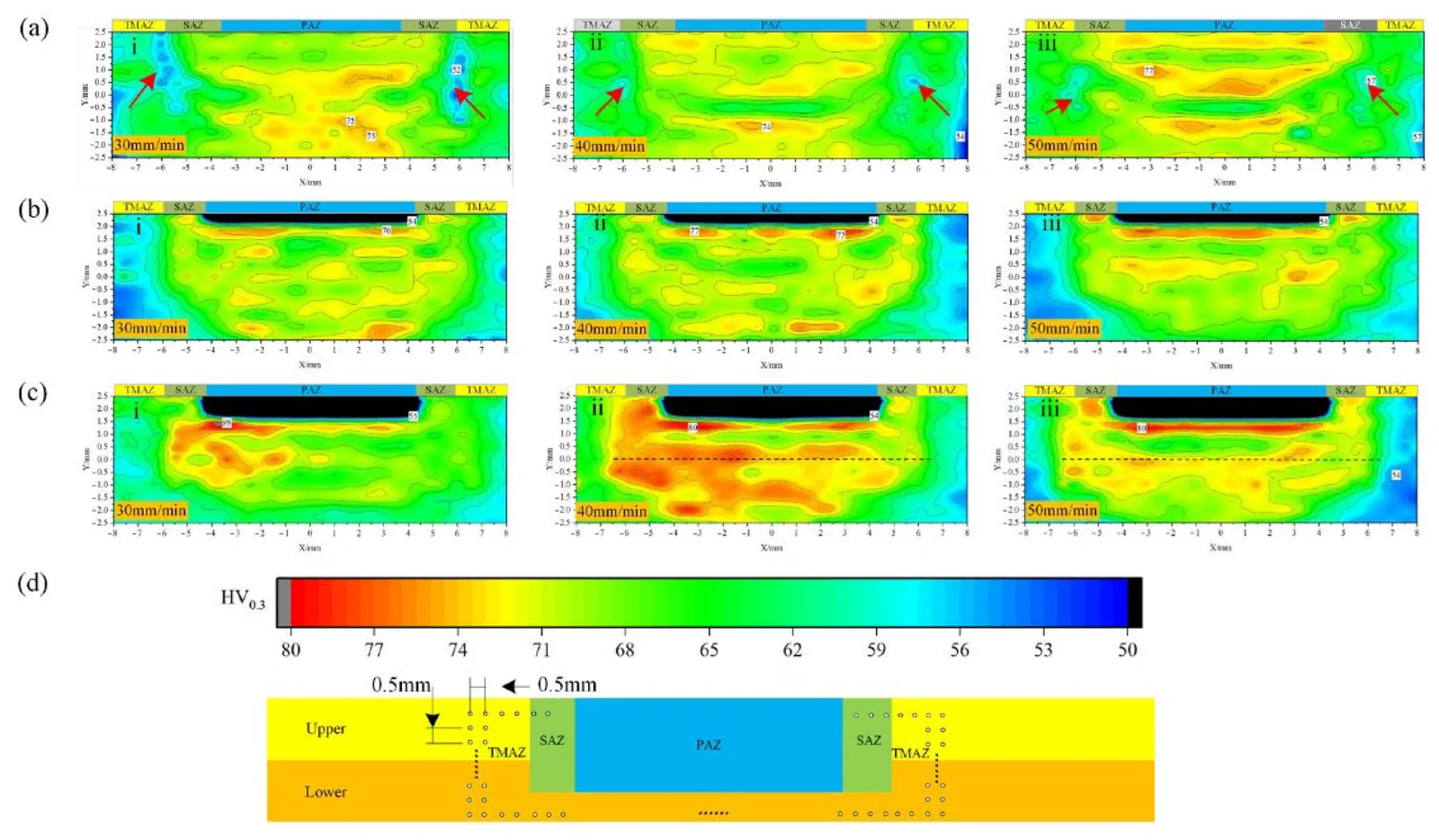
Microhardness distribution in weld zone under different process parameters, (**a**) PDSP = 0, (**b**) PDSP = 0.5 mm, (**c**) PDSP = 1 mm. (**d**) Microhardness test locations and corresponding scale (welding speed: i = 30 mm/min, ii = 40 mm/min, iii = 50 mm/min) [52].

Jiang et al. [52] found that the plunging depth of stirring pin (PDSP) was conducive to bolstering the weld zone’s overall performance. However, excessive PDSP gives rise to deep grooves in the PAZ, which may abate the load-bearing capacity of the joint. At the same time, when PDSP = 1 mm, greater axial pressure can result in better microhardness, but the microhardness of the spot weld zone gradually decreases as the welding speed increases, eventually falling below that of PDSP = 0.5 mm, as shown in Figure 11. Zhang et al. [53] performed friction stir welding tests on aluminum alloy plates with thicknesses of 2.5 mm and 25 mm. The results indicated that the stirring pin with an enlarged end structure can effectively enhance the material flowability in the welding zone, promote sufficient mixing at the lap interface, and eliminate the inherent hook defects of friction stir welding, thereby significantly improving the mechanical performance of joints, with a maximum failure load of 7.97 kN. Gibson et al. [54] explored the influence of shoulder surface morphology on the mechanical properties of friction stir lap welding (FSLW) joints. It was verified that the stepped shoulder can achieve optimal process matching and obtain a larger effective lap width under low rotational and welding speeds.

FSW involves complex material flow behavior during the welding process. Microstructure evolution such as grain size, grain boundary orientation and second-phase particle distribution in each region of the weld has a significant effect on the mechanical properties of FSW joints. Relying on the mechanical action generated by the stirring head and the plastic flow of the material, aluminum alloy can achieve efficient solid-state connection. However, the application of FSW in electronic precision equipment is subject to significant spatial constraints: the welding process requires the tool shoulder to apply hundreds to thousands of kilograms of axial pressure and relies on a sufficiently wide lap area to carry the force, while the highly integrated electronic precision equipment often does not have the geometric space to accommodate the movement trajectory of the stirring head and the back support structure. Spatial accessibility alone restricts its use in highly integrated precision equipment. Whether the inherent space limitations of electronic precision equipment can be coordinated with the requirements of FSW is still a problem to be solved—it may be necessary to fundamentally design a new tool driver architecture, rather than incrementally modify the existing friction stir welding variants.

## 5. Laser-Arc Hybrid Welding

The high integration of aluminum alloy electronic equipment leads to an increasingly tight welding space. The continuous improvement of equipment accuracy makes the deformation control requirements more stringent. The trend of lightweight makes the requirements for mechanical properties of load-bearing components continue to increase. After the weight reduction design, the high-load turntable needs to use high-strength and high-toughness materials to ensure the rigidity and strength. However, the characteristics of the material itself make it difficult for the traditional welding technology to meet the reliability requirements. It is necessary to meet the precision high-strength connection through laser-arc hybrid welding.

Laser-arc hybrid welding (LAHW) consists of two heat sources: high-energy-density laser beam and arc, which is not a simple superposition of heat sources. During the welding process, there is a strong interaction between the metal vapor emitted from the keyhole under the action of laser and the arc plasma generated by arc discharge, which has a great influence on the arc shape and droplet transfer. Therefore, the arc-droplet behavior of laser-arc hybrid welding is the focus of hybrid welding research. In the process of laser-arc hybrid welding, it is generally believed that the laser attracts and compresses the arc under the action of the laser to improve the stability of the arc. Li Xuwen et al. [55] pointed out that the addition of laser has an attraction and compression effect on the arc, reducing the degree of arc voltage fluctuation. Liu Xiyang et al. [56] found that the laser can reduce the drift of the arc action point and improve the arc stability. In a study of the laser--TIG hybrid welding process, Mu et al. [57] found that the laser promoted the significant oscillation of the arc tail under the action of the laser, pointing out that this phenomenon was caused by the high-speed oscillating metal vapor ejected from the keyhole, and found that the metal vapor can promote the arc contraction through physical shielding. Zhang et al. [58] studied the arc-droplet behavior during fiber laser-arc hybrid welding based on a high-speed camera system and found that the stability of the welding process would be destroyed under high arc voltage and short wire spacing. Relevant scholars have pointed out that the distance between the filaments is the main factor affecting the laser-arc coupling. If the distance between the filaments is too small, the laser interferes with the droplet transfer. In addition, the laser is easy to irradiate the droplet surface and hinder the beam transmission. In the study of narrow gap laser-arc hybrid welding, Wang et al. [59] pointed out that the arc behavior is closely related to the dynamic behavior of the droplet at the end of the wire, and the unstable swing of the molten metal at the end of the wire is easy to cause the arc deflection.

**Figure 12:**
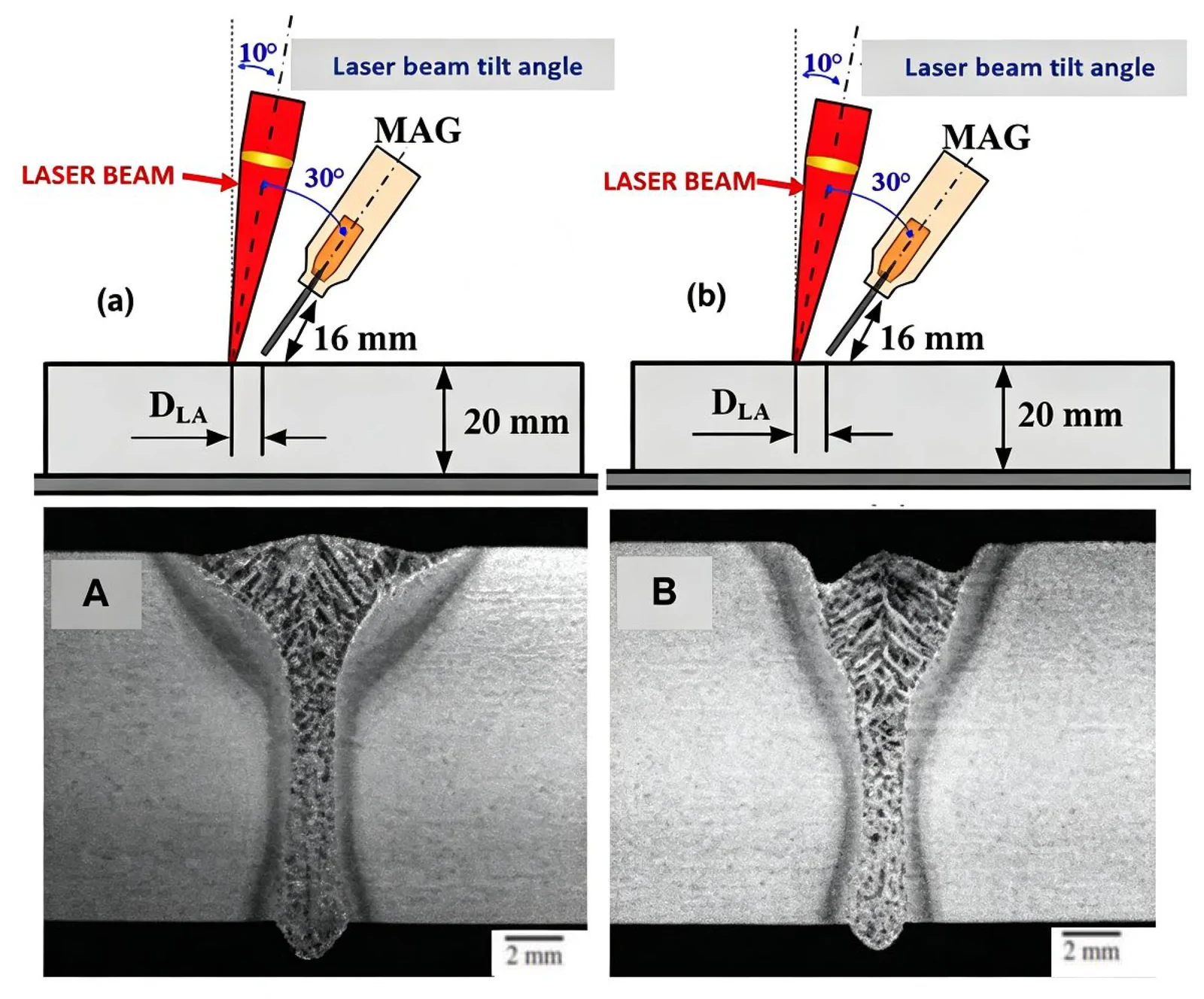
The schematic diagram of LAHW process and welded joint macrostructures. (**a**) arc leading mode; (**b**) laser leading mode [62].

The change in heat source guidance mode will affect the arc morphology, droplet transfer and molten pool flow and then change the weld forming quality. Zhu et al. [60] studied the influence of heat source guidance mode on weld surface formation, porosity, weld width/penetration, weld pool--keyhole dynamic behavior, etc. and pointed out that under low power and high welding speed, the penetration depth under arc guidance mode is larger. At high power and low welding speed, the penetration depth under laser guidance can be increased by about 20% compared with that under arc guidance. Liu et al. [61] pointed out that under the guidance of arc, the arc force, that is, the droplet impact force, will make the laser beam act on the slope of the molten pool, promote the flow of molten metal to the bottom of the molten pool, and increase the penetration depth of the laser-arc coupling zone. When the laser precedes the electric arc, the cross-sectional area of plasma expands, which reduces arc resistance and arc voltage and stabilizes the welding process. Under this condition, droplet transfer occurs in the spray mode, and the molten pool presents bullet-like morphology. In contrast, when the laser is placed behind the arc, the ionization degree of metal vapor is low, leading to the contraction of thermal plasma. This extends the droplet formation time, weakens droplet transfer, and produces a gourd-shaped molten pool, as shown in Figure 12.

**Figure 13:**
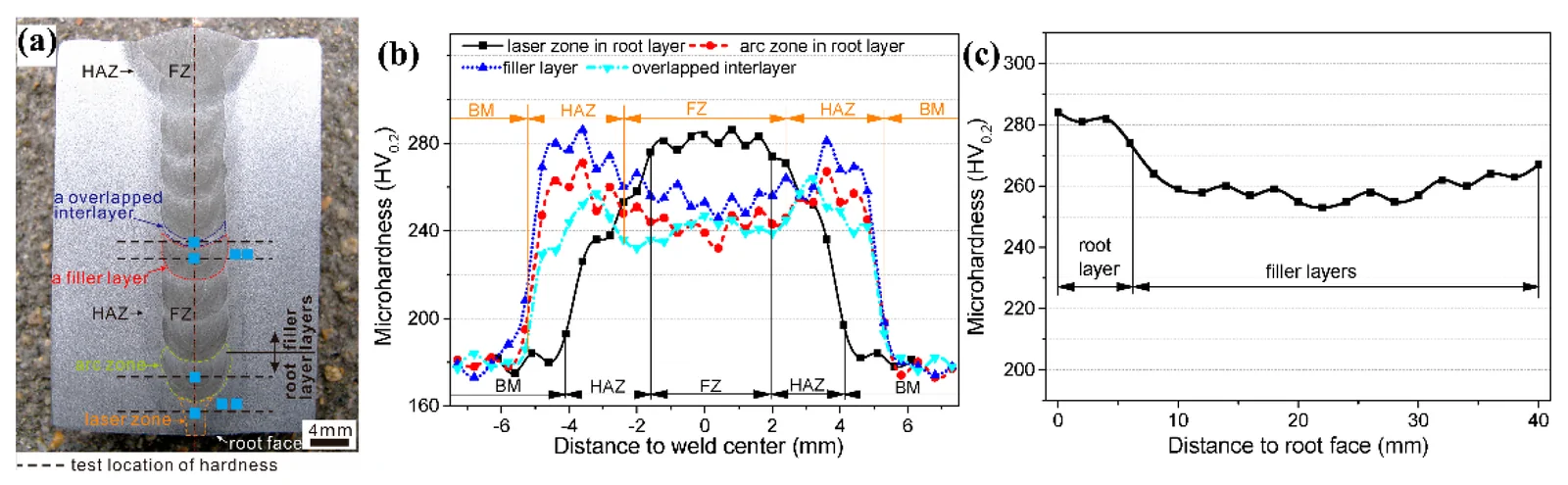
(**a**) Cross-section of 40 mm thick joint created with narrow gap LAHW and microhardness profiles across weld surface: (**b**) profiles along horizon direction of different layers; (**c**) profile along thickness direction of weld center [65].

Ascari et al. [63] investigated the welding procedures of AA6082 aluminum alloy with a 3 kW laser--MIG hybrid heat source and characterized pore features under various welding modes. Their results revealed that arc current exerts an effect on the porosity in the fusion zone under the short-circuit transfer mode, as well as the average pore size in both short-circuit transfer and pulsed arc transfer modes. In addition, variations in laser power and arc separation distance impose negligible impacts on porosity and average pore diameter. Gao et al. [64] carried out welding experiments on ultra-fine grained steel using a high-power laser-TIG hybrid heat source and systematically analyzed the effects of welding parameters on weld geometry, microstructure morphology, grain growth in the heat-affected zone (HAZ), and mechanical properties. The study demonstrated that increasing the laser-to-arc energy ratio reduces weld width and inhibits grain coarsening of ferrite and grain growth within the HAZ. A distinct softened zone emerges in the HAZ when the laser-to-arc energy ratio is low. By contrast, no softened zone forms in the HAZ at a high laser-to-arc energy ratio owing to the low heat input. Zhang et al. [65] fabricated welded joints of 40 mm thick mild steel by narrow gap LAHW. The lower part of the weld has the lowest mechanical properties due to the lowest amount of acicular ferrite, but its hardness is 49% higher than that of base metal. The microhardness deviation of all filler layers along weld thickness direction is no more than 15 HV0.2, indicating that no temper softening appeared during multiple heat cycles, as shown in Figure 13c. Song et al. [66] fabricated welded joints of 7075 aluminum alloy via high-power oscillating laser-arc hybrid welding, achieving a tensile strength of approximately 420 MPa. Furthermore, the high-frequency laser-assisted process effectively optimized residual stress distribution and extends the fatigue life of welded joints.

The above research shows that the heating of laser in laser-arc hybrid welding can change the distribution of heat source, attract and compress the arc, improve the stability of arc, change the force of droplet and improve the stability of droplet transfer. By adjusting the matching parameters of high power laser and arc heat source, the stability and energy density of hybrid heat source welding can be improved, and the welding penetration can be increased. Optimizing the process based on the experimental results can reduce the welding defects under different welding conditions (joint structure, welding speed and environment), improve the welding quality, and solve a large number of practical engineering welding problems. In addition, laser-arc hybrid welding has achieved reliable connection of 7075 high-strength aluminum alloy, and the tensile strength of the welded joint can reach 420 MPa, which is significantly higher than 230 MPa of the welded joint of traditional commonly used aluminum alloy 5xxx aluminum. This comparison is design-driven rather than generic: because the wall thickness of the load-bearing skeleton is governed by the absolute strength of the welded joint (the limiting cross-section), the-1.8-fold strength advantage of 7075 directly enables a corresponding reduction in wall thickness. It should be emphasized that 7075 is otherwise difficult to join by electron beam welding owing to hot cracking and Zn/Mg evaporation (Section 3). With this strength advantage, structural parts made of 7075 can substantially reduce wall thickness under the same load-bearing requirements: the weight of the turntable is directly reduced, and the released space and weight allowance permit the integration of more electronic precision devices, further improving the overall performance of the system.

## 6. Discussion

This section compares the four processes (Brazing, EBW, FSW, and LAHW) under a unified framework of physical limitations and a criterion-based summary table in order to support the selection of an appropriate joining technology.

### 6.1. Unified Classification of Physical Limitations

Thermodynamic constraint: The process window of vacuum brazing of aluminum alloy is limited by the phase-diagram relationship between the matrix and the solder. The solidus temperature (515–545 °C) of die-cast aluminum alloys (ADC12, A380) is lower than the melting point of the Al-Si eutectic solder (about 577 °C), whereas the actual brazing process temperature needs to reach 590–610 °C, so that the substrate enters the local melting state before the solder is fully wetted. This thermodynamic deadlock cannot be overcome by adjusting the process parameters.

Material-intrinsic constraint: the vacuum-volatilization problem exists in EBW of 7xxx-series aluminum alloys. The high vapor pressure of Zn and Mg in vacuum leads to their selective volatilization during welding, which shifts the weld composition, increases the hot-cracking sensitivity, and softens the joint. Systematic research on this problem is still insufficient, and it constitutes the main obstacle to the engineering application of this alloy class.

Spatial-geometric constraint: FSW faces the restriction of insufficient welding space. The strength of FSW joints can reach more than 80% of the base metal, and the cost is only about 8–12% of that of electron beam welding (on a per-meter processing basis). However, the process depends on the large axial pressure applied by the stirring tool and on a sufficiently wide lap area to bear this pressure; the highly integrated geometric space inside electronic precision equipment can hardly accommodate the tool trajectory and the back-support structure.

Breaking the coupling: the breakthrough and potential of laser-arc hybrid welding. Laser-arc hybrid welding achieves a joint tensile strength of about 420 MPa in 7075 aluminum alloy, substantially higher than the about 230 MPa of the conventional 5xxx aluminum. Because the wall thickness of the load-bearing skeleton is governed by the absolute strength of the welded joint, this strength advantage enables a corresponding reduction in wall thickness, so that 7075 structural parts can be made thinner and lighter, releasing space and weight allowance for the integration of more electronic precision devices.

### 6.2. Comparison Table and Criterion Discussion

Table 1 summarizes the four processes across seven criteria. Of these, applicable materials (i) and joint strength (vi) are literature- and standard-based; the remaining criteria summarize the authors’ engineering practice and are identified as such. (i) Applicable materials correspond to the alloys discussed in Section 2, Section 3, Section 4 and Section 5. (ii) Welding cost: Vacuum brazing is a batch furnace process and is therefore quoted per unit area (RMB/m^2^), whereas electron beam welding, friction stir welding, and laser-arc hybrid welding are trajectory-guided and quoted per unit length (RMB/m); the two bases are not directly interchangeable. The statement “FSW ≈ 8–12% of EBW” refers to the per-meter processing cost for comparable joint lengths (excluding workpiece-specific tooling amortization). (iii) Typical size requirements list the component-size ranges encountered in electronic precision equipment. (iv) Design space requirements describe the geometric clearance needed for the tooling. (v) Joint configuration lists the predominant joint types. (vi) Joint strength requirement is the joint strength relative to the base metal, according to GJB 294A II welds for FSW, EBW, and LAHW. (vii) Welding deformation requirement lists the deformation allowance, which is set to the process capability limit (the design deformation allowance equals the achievable deformation of the selected process) under the stated conditions (e.g., flatness of FSW structure after post-weld annealing).

### 6.3. Coupling of Constraints and Outlook

These four points reveal a common pattern: the engineering bottleneck is not a lack of joining capability but the coupling of constraints across different categories. The metallurgical facts—the hot-cracking susceptibility of 7075 and the selective evaporation of Zn and Mg during welding—are well established in the literature [67]. On this basis, the authors’ engineering practice indicates that, for the 7075 skeleton, the strict flatness requirement (1 mm/m) imposes heavy fixturing (a spatial-geometric constraint) that, together with these material-intrinsic constraints, produces the residual-stress-driven cracking currently preventing the substitution of 7075 aluminum for 5xxx aluminum. Breaking this coupling—by relaxing the spatial constraint through new tooling/process architectures or by suppressing the material constraint through heat-source modulation (exemplified by oscillating laser-arc hybrid welding, which achieves −420 MPa in 7075 aluminum [66])—is the central direction for future work. Likewise, the thermodynamic deadlock of brazing die-cast aluminum and the thermal-expansion mismatch of metal/ceramic packages point to material-system and filler-design solutions. Future advances will thus come not from incremental process optimization within a single constraint category, but from strategies that simultaneously decouple the three constraint categories.

## 7. Conclusions

Electronic precision equipment comprises two structure tiers: the load-bearing skeleton, whose main structural material is the 5xxx aluminum alloy (with 7075 aluminum as the target for future lightweight designs), and the precision functional devices (waveguides, sub-arrays, antenna horns), which are predominantly 6xxx-series alloys such as 6061/6063. The four joining technologies reviewed here—vacuum brazing, electron beam welding, friction stir welding, and laser-arc hybrid welding—each serve these tiers with distinct advantages and limitations, which are classified into three coupled constraint categories in Section 6.

None of the four technologies is universally applicable: each is limited by a distinct combination of thermodynamic, material-intrinsic, and spatial-geometric constraints, and it is the coupling of these constraints—rather than any single limitation—that constitutes the current engineering bottleneck. Breaking this coupling is the central direction for future work, with oscillating laser-arc hybrid welding of 7075 aluminum exemplifying a first step toward lightweight, high-reliability structures.

## Figures and Tables

**Table 1:** Multi-dimensional summary of different welding processes.

	Welding Methods			
	Brazing	EBW	FSW	LAHW
Applicable materials	6xxx Al; Cu/dissimilar	5xxx Al, 6xxx Al (with filler wire), structural steel	5xxx Al, 6xxx Al, 7xxx Al	Steel; 5xxx Al, 6xxx Al, 7xxx Al
Welding cost	Per furnace load, −10^3^ RMB/m^2^	−10^2^ RMB/m	−10^1^ RMB/m	−10^1^ RMB/m
Typical size requirements	≤2 m	Depends on chamber size	0.4–4 m	≥4 m
Design space requirements	No lap step required	Compact space; lap step close to 1 mm	Weld depth/lap step ≈ 1:1	Tooling space and hybrid torch space required
Joint configuration	Lap	Butt	Butt	Butt; filet
Joint strength requirement	30% of base metal [30]	80% of base metal	80% of base metal	80% of base metal
Welding deformation requirement	0.3 mm per 1 m	0.5 mm per 1 m	0.8 mm per 1 m	1 mm per 1 m

Footnote: Vacuum brazing is a batch furnace process and is quoted per unit area (RMB/m^2^); EBW, FSW, and LAHW are trajectory-guided and are quoted per unit length (RMB/m). The two bases are therefore not directly interchangeable. The statement ‘FSW ≈ 8–12% of EBW’ refers to the per-meter processing cost for comparable joint lengths (excluding workpiece-specific tooling amortization).

## Data Availability

The data presented in this study will be available from the corresponding author upon reasonable request.
